# Analysis of Expression Patterns of MicroRNAs That Are Closely Associated With Renal Carcinogenesis

**DOI:** 10.3389/fonc.2019.00431

**Published:** 2019-05-31

**Authors:** Ei Shiomi, Tamotsu Sugai, Kazuyuki Ishida, Mitsumasa Osakabe, Takashi Tsuyukubo, Yoichiro Kato, Ryo Takata, Wataru Obara

**Affiliations:** ^1^Department of Molecular Diagnostic Pathology, School of Medicine, Iwate Medical University, Morioka, Japan; ^2^Department of Urology, School of Medicine, Iwate Medical University, Morioka, Japan

**Keywords:** clear cell renal cell carcinoma, cluster analysis, microRNA-135a-5p, microRNA, renal carcinogenesis

## Abstract

**Background:** MicroRNAs (miRNA) are frequently dysregulated in clear cell renal cell carcinoma (ccRCC).

**Objective:** This study aimed to elucidate the role of miRNA expression patterns in renal carcinogenesis and to identify the specific miRNAs that exhibit expression patterns closely associated with patient outcomes.

**Methods:** We examined the expression patterns of selected miRNAs, including miRNA-155-5p, miRNA-122-5p, miRNA-21-5p, miRNA-185-5p, miRNA-106a-5p, miRNA-106b-3p, miRNA-34b-3p, miRNA-210-3p, miRNA-141-3p, miRNA-200c-3p, miRNA-135a-5p, miRNA-30a-5p, miRNA-218-5p, miRNA-429, miRNA-200a-3p and miRNA-200b-3p, in 96 samples of ccRCCs using the TaqMan real-time PCR method. In addition, cluster analysis was performed to stratify expression patterns of multiple miRNAs.

**Results:** In the present study, three distinct subgroups could be clearly stratified in ccRCCs. Subgroup 1 was characterized by upregulation of miRNA-155-5p, miRNA-122-5p, miRNA-21-5p, miRNA-185-5p, miRNA-106a-5p, miRNA-106b-3p, miRNA-34b-3p and miRNA-210-3p. Subgroup 2 was closely associated with downregulation of miRNA-141-3p, miRNA200c-3p, miRNA-30a-5p, miRNA-218-5p, miRNA-429, miRNA-200a-3p and miRNA-200b-3p. Moreover, significant lower expression of miRNA-135a-5p was a distinctive feature of subgroup 3, which was correlated with metachronous metastasis. Among the individual markers in subgroup 3, miRNA-135a-5p was retained in multivariate analysis. The cutoff value of miRNA-135a-5p expression to identify the association of an altered level of miRNA-135a-5p with metachronous metastasis in ccRCCs was determined and showed excellent specificity.

**Conclusion:** We suggest that the expression pattern of the chosen miRNAs is useful to identify renal carcinogenesis and to help identify the association of such expression patterns with metachronous metastasis in ccRCCs. In addition, miRNA-135a-5p was an excellent marker for prediction of metachronous metastasis.

## Introduction

Renal cell carcinoma (RCC) is the most common histological type of urological cancer. It constitutes fewer than 3% of all malignant epithelial neoplasms (1, 2). RCC is a potentially curable disease, especially if invasive and metastatic spreads have not occurred prior to diagnosis (3). However, approximately 40% of patients with RCC die due to disease progression (metastasis). Thus, this tumor is a particularly lethal malignant urological tumor (4). RCC is also a heterogeneous disease in terms of histological features (5). The histological classification of RCCs is extremely important, given that such classification might help determine the subtype, prognosis and treatment of the disease (5). However, histological classification is insufficient for prediction of patient outcome in RCC, given that it cannot predict the course of disease even in clear cell RCC (ccRCC), which is the most common histological type (6). Recently, biological markers that predict patient outcome have been developed in RCC (7). However, some of those biomarkers predicting patient outcome or follow-up of RCC might not be available (7). Therefore, investigators must continue to seek novel tumor biomarkers that predict the precise outcome for patients with RCC (8).

MicroRNAs (miRNAs) are small non-coding RNAs that are 20–22 nucleotides in length. They regulate gene expression at the post-transcriptional level by binding to miRNA-specific sequences (9). miRNAs have several functions, including the regulation of tumor cell proliferation, apoptosis, invasion and tumor vascular formation. Regulation is achieved through modulating the expression of specific target genes (10). Moreover, miRNAs can be aberrantly expressed in cancer, suggesting that miRNAs can be oncogenic or cancer suppressive (11). Recent studies have shown that aberrant miRNA expression is associated with overall patient survival, tumor stage and the development of metastases and recurrences (3). Accumulating evidence shows that miRNAs constitute promising biomarkers for accurate prediction of patient outcome as well as targets for more efficient treatment (12).

Many studies of biological makers, including miRNAs, have been reported in RCCs (7). Comprehensive genome-wide analyses of miRNA expression have identified differences in expression levels between tumors and normal tissues (13–15). Importantly, miRNA expression datasets may contain inconsistent results due to choice of technological platforms, differences in patient populations and small sample sizes (16). Moreover, in many previous studies, a single miRNA has been associated with patient outcome in ccRCC (17, 18). However, an individual marker of miRNA might fail to identify its role in clinical management of RCC.

In the present study, we attempted to achieve the following: (1) define an integrated miRNA expression profile for comparison of RCC vs. normal tissue, (2) identify possible associations between miRNA expression patterns and clinical information, such as metachronous metastasis, and (3) test the potential clinical usefulness of individual miRNAs as prognostic and predictive biomarkers in ccRCC.

## Patients

Ninety-six paired specimens of cancer tissues and adjacent non-cancerous tissues were obtained from 96 ccRCC patients who underwent surgery at Iwate Medical University Hospital. The fresh tissues were frozen in liquid nitrogen immediately after dissection. All tissue samples were confirmed to be ccRCC type based upon their pathology and they were classified according to the WHO guidelines for tumors of the urinary system and male genital organs (19) with a slight modification. The clinicopathological findings are shown in Table 1 and include sex, age, tumor size, tumor location, tumor nuclear grade, Fuhrman grade, venous invasion, TNM stage, and presence of metachronous metastasis according to the Japanese Classification for Renal Cell Carcinoma. The median duration of follow-up of metachronous metastasis was 37 months (range, 11–57 months). During this follow-up period, three patients with metachronous metastasis died.

**Table 1:** Clinicopathologic findings of clear cell renal cell carcinoma we examined.

**Findings**		**Cases (%)**
Total		96
Sex	Men	63 (65.6)
	Women	33 (34.4)
Age (year)	Range (median)	32–88 (66)
Size (mm)	Range (median)	15–125 (45)
Locus	Right	52 (54.2)
	Left	44 (45.8)
Fuhrman grade	Grade 2	62 (64.5)
	Grade 3	34 (35.5)
Necrosis	Presence	20 (20.8)
Venous invasion	Positive	21 (21.8)
pT stage	pT1	61 (63.5)
	pT2	13 (13.5)
	pT3	22 (22.9)
Stage	I	61 (63.5)
	II	13 (13.5)
	III	22 (22.9)
Metachronous metastasis	Positive	22 (22.9)

Written informed consent regarding tissue specimens for research purposes was obtained in each case. This study was approved by the Iwate Medical University (HGH29-25) and was conducted in accordance with the Helsinki Declaration.

## RNA Isolation

Fresh tumor tissue was obtained from surgically dissected tissue lacking necrosis. Normal tissues (distant from the tumor) were used as controls. All tissue samples were stored at −80°C until RNA extraction. Starting with both fresh tumor tissue and the corresponding normal tissue, miRNA was extracted using the mirVana™ miRNA Isolation kit (Thermo Fisher Scientific, Inc.) according to the manufacturer's instructions. The quantity and quality of RNA were evaluated by a BioPhotometer and RNA integrity was determined by gel analysis.

## miRNAs Evaluated

In the present study, the following miRNAs were evaluated: miRNA-155-5p (17), miRNA-122-5p (18), miRNA-21-5p (20), miRNA-185-3p (21), miRNA-106a-5p (22), miRNA-106b-3p (23), miRNA-34b-3p (24), miRNA-210-3p (25), miRNA-141-3p (26), miRNA-200c-3p (27), miRNA-135a-5p (28), miRNA-30a-5p (29), miRNA-218-5p (30), miRNA-429 (31), miRNA-200a-3p (32) and miRNA-200b-3p (32), all of which are expressed in ccRCC. Primer sequences are shown in Table 2.

**Table 2:** List of miRNA primers used.

**miRNA**	**Sequence (5′-3′)**
hsa-miRNA-155-5p	UUAAUGCUAAUCGUGAUAGGGGU
hsa-miRNA-122-5p	UGGAGUGUGACAAUGGUGUUUG
hsa-miRNA-21-5p	UAGCUUAUCAGACUGAUGUUGA
hsa-miRNA-185-3p	AGGGGCUGGCUUUCCUCUGGUC
hsa-miRNA-106a-5p	AAAAGUGCUUACAGUGCAGGUAG
hsa-miRNA-106b-3p	CCGCACUGUGGGUACUUGCUGC
hsa-miRNA-34b-3p	CAAUCACUAACUCCACUGCCAU
hsa-miRNA-210-3p	CUGUGCGUGUGACAGCGGCUGA
hsa-miRNA-141-3p	UAACACUGUCUGGUAAAGAUGG
hsa-miRNA-200c-3p	UAAUACUGCCGGGUAAUGAUGGA
hsa-miRNA-135a-5p	UAUGGCUUUUUAUUCCUAUGUGA
hsa-miRNA-30a-5p	UGUAAACAUCCUCGACUGGAAG
hsa-miRNA-218-5p	UUGUGCUUGAUCUAACCAUGU
hsa-miRNA-429	UAAUACUGUCUGGUAAAACCGU
hsa-miRNA-200a-3p	UAACACUGUCUGGUAACGAUGU
hsa-miRNA-200b-3p	UAAUACUGCCUGGUAAUGAUGA

## Quantitative Real-time PCR

Detection and quantification of mature miRNAs was achieved by conducting reverse transcription quantitative real-time PCR (RT-qPCR) in conjunction with the TaqMan miRNA Assay (Applied Biosystems, Foster City, CA) according to the manufacturer's protocol. Triplicate RNA samples were used. Reverse transcription of RNA to cDNA was achieved by using the TaqMan MicroRNA Reverse Transcription Kit (Applied Biosystems). Reverse transcription reactions used a Gene Amp PCR system 9,700 thermal cycler (Applied Biosystems). Samples were incubated at 16°C for 30 min, 42°C for 30 min, and 85°C for 5 min. We included an RT-negative control in each set of reactions. The reaction mix (20 μL final volume) contained the RT product, TaqMan 2X Universal PCR Master Mix II and the appropriate 20X MicroRNA Assay Mix, including the specific probe for the miRNA of interest. PCR reactions, including *RNU6B* (endogenous control) utilized the StepOnePlus Real-Time PCR Systems (Applied Biosystems, San Diego, CA) and the following reaction conditions: 10 min at 95°C and 40 cycles of 15 s at 95°C, 60 s at 60°C. Inter-assay controls and calibrators were included in each 96-well plate. All TaqMan assays were run in triplicate using an AB StepOnePlus Real-Time PCR System. Ct values were calculated using StepOne Software v2.2.2 with automatic baseline settings. RNU6B (assay ID: 001093) was used as an endogenous control for normalizing the expression levels of miRNAs. The mean Ct values were subtracted from the corresponding Ct value for the examined miRNAs resulting in the ΔCt value that was used for relative quantification of miRNA expression (ΔΔCt method). Changes in expression levels in tumor samples are shown as relative (fold-change) to normal tissue.

### Hierarchical Analysis of the Expression of miRNA Markers

We conducted hierarchical cluster analysis to group the samples according to their quantitative levels. This maximized homogeneity for each group and assured the greatest differences between the groups. This was achieved with open-access clustering software (Cluster 3.0 software; bonsai.hgc.jp/~mdehoon/software/cluster/software.htm). The clustering algorithm was set to centroid linkage clustering, which is the standard hierarchical clustering method used in biological studies.

## Statistical Analysis

Differences in the clinicopathological variables including sex, age, tumor size, tumor location, tumor nuclear grading, Fuhrman grade, venous invasion, TNM stage and presence of metachronous metastasis among the subgroups were analyzed using chi-square tests in Stat Mate-III (Atom, Tokyo, Japan). If a significant statistical difference among the subgroups was identified, further chi-square tests between 2 specific groups were performed. Differences in the age distribution and ages among the groups were evaluated using the Kruskal-Wallis H test in Stat Mate-III. A *p* < 0.05 was considered to indicate significance.

We calculated disease-free survival (without metachronous metastasis) of the patients based upon the date of the surgery and the date of the last follow-up or patient metachronous metastasis. The Cox proportional hazards regression model was used for univariate and multivariate survival analyses. The level of significance was accepted at *P* < 0.05, and the confidence interval (CI) was determined at the 95% level. Statistical analyses were conducted with the JMP 10.0 software package (SAS Institute, Inc., Cary, NC, USA) for Windows.

## Results

### Hierarchical Clustering Based on Dysregulated miRNA Expression

**Figure 1:**
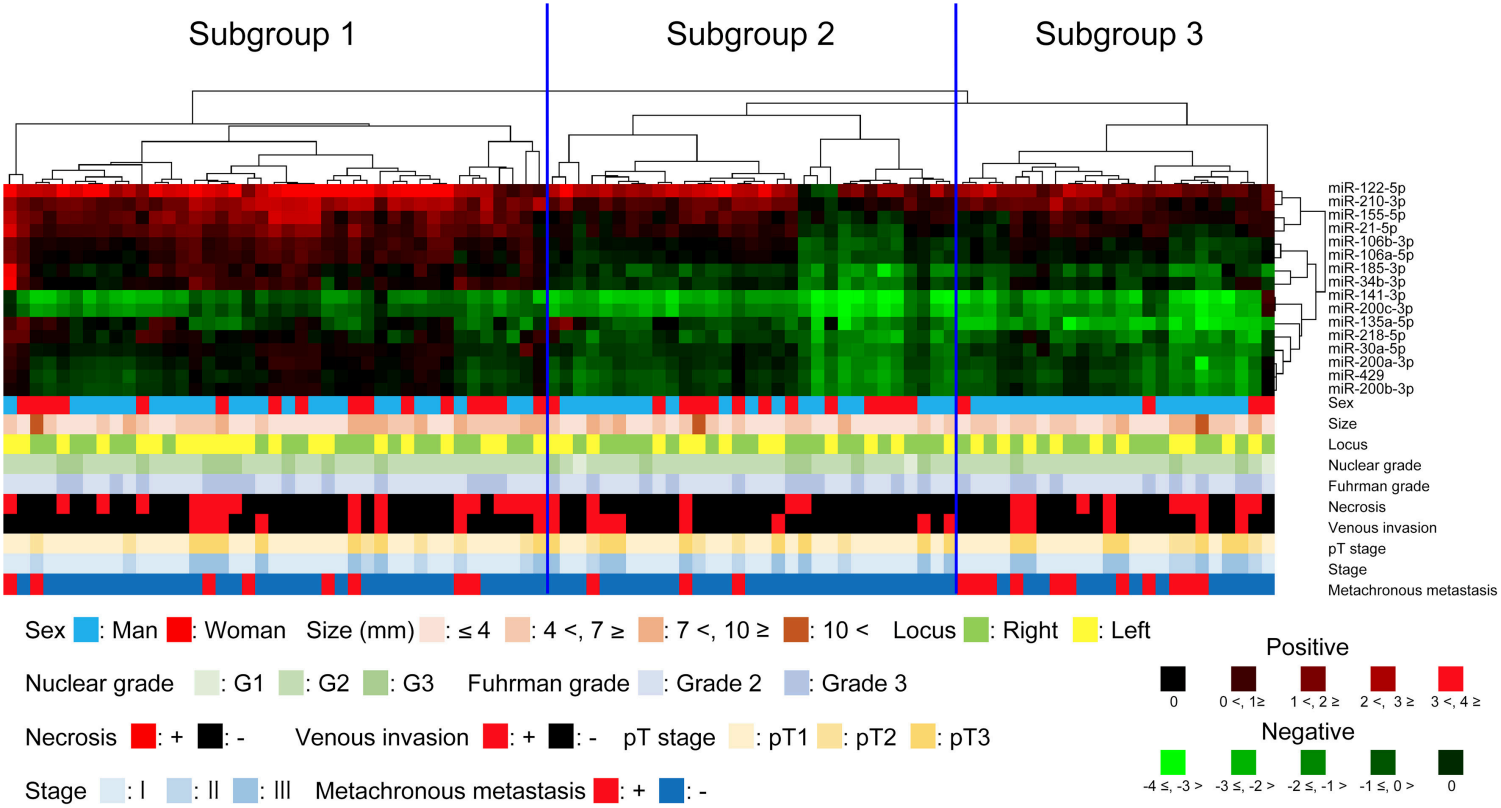
Hierarchical cluster analysis of clear cell renal cell carcinoma based on microRNA expression patterns. The clear cell renal cell carcinoma samples were classified into three subgroups.

We examined the expression levels of miRNAs and performed miRNA hierarchical clustering. As a result, we identified three distinct subgroups (Figure 1), in which the expression of each miRNA marker in tumor tissue is indicated by the vertical line, and the horizontal lines denote “relatedness” between samples.

#### Differences in the Clinicopathological Findings Among Subgroups 1, 2, and 3

No statistical differences in clinicopathological findings were found among the subgroups, including sex, age, tumor size, tumor location, tumor nuclear grading, Fuhrman grade, venous invasion and TNM stage. Nonetheless, we found that the frequency of metachronous metastasis was greater in subgroup 3 than in subgroups 1 and 2 (subgroup 2 vs. 3, *P* = 0.006), though such frequency between subgroup 1 and 3 did not reach statistical level (*P* = 0.077) (Table 3).

**Table 3:** Clinicopathologic findings of clear cell renal cell carcinomas based on each subgroup.

**Findings**		**Subgroup 1 (%)**	**Subgroup 2 (%)**	**Subgroup 3 (%)**	***P-*value**
Total		41	31	24	
Sex	Men	25 (60.9)	18 (58.0)	20 (83.4)	0.085
	Women	16 (39.1)	13 (42.0)	4 (16.6)	
Age (year)	Range (median)	32–81 (66)	34–88 (70)	51–81 (65)	0.296
Size (mm)	Range (median)	18–110 (50)	15–110 (40)	22–125 (50)	0.289
Locus	Right	20 (48.8)	18 (58.1)	14 (58.3)	0.658
	Left	21 (51.2)	13 (41.9)	10 (41.7)	
Fuhrman grade	Grade 2	27 (65.9)	20 (64.5)	15 (62.5)	0.963
	Grade 3	14 (34.1)	11 (35.5)	9 (37.5)	
Necrosis	Presence	6 (14.6)	5 (16.1)	9 (37.5)	0.072
Venous invasion	Positive	8 (19.5)	8 (25.8)	5 (20.8)	0.809
pT stage	pT1	28 (68.3)	19 (61.3)	14 (58.3)	0.828
	pT2	6 (14.6)	4 (12.9)	3 (12.5)	
	pT3	7 (17.1)	8 (25.8)	7 (29.2)	
Stage	I	28 (68.3)	19 (61.3)	14 (58.3)	0.828
	II	6 (14.6)	4 (12.9)	3 (12.5)	
	III	7 (17.1)	8 (25.8)	7 (29.2)	
Metachronous metastasis	Positive	8 (19.5)^a^	3 (9.7)^b^	11 (45.8)	0.006, 0.077^a^, 0.006^b^

*P < 0.05 was considered significant*.

a *Sub1 vs. Sub3*.

b *Sub2 vs. Sub3*.

#### Differences in the Dysregulation of miRNAs Among Subgroups 1, 2, and 3

**Figure 2:**
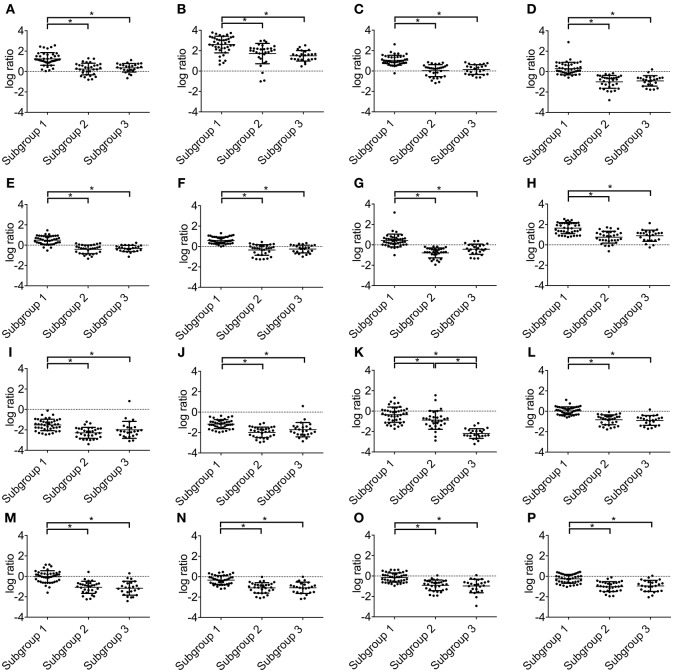
Expression levels of microRNAs in subgroups 1, 2 and 3. **(A)** miRNA-155-5p; **(B)** miRNA-122-5p; **(C)** miRNA-21-5p, **(D)** miRNA-185-3p; **(E)** miRNA-106a-5p; **(F)** miRNA-106b-3p; **(G)** miRNA-34b-3p; **(H)** miRNA-210-3p; **(I)** miRNA-141-3p; **(J)** miRNA-200c-3p; **(K)** miRNA-135a-5p; **(L)** miRNA-30a-5p; **(M)** miRNA-218-5p; **(N)** miRNA-429; **(O)** miRNA-200a-3p; **(P)** miRNA-200b-3p.

Statistically significant differences were found in the upregulation of miRNA-155-5p, miRNA-122-5p, miRNA-21-5p, miRNA-185-3p, miRNA-106a-5p, miRNA-106b-3p, miRNA-34b-3p and miRNA-210-3p between subgroups 1 and 2 and between subgroups 1 and 3. Moreover, there were significant differences in the downregulation of miRNA-141-3p, miRNA-200c-3p, miRNA-30a-5p, miRNA-218-5p, miRNA-429, miRNA-200a-3p and miRNA-200b-3p between subgroups 1 and 2 and between subgroups 1 and 3. However, significantly lower expression of miRNA-135a-5p was found among subgroup 1 and 2, subgroup 2 and 3 and subgroup 1 and 3. Such association was observed in miRNA-135a-5p alone. Therefore, significantly lower expression of miRNA-135a-5p was a distinctive feature of subgroup 3. These data are presented in Figure 2.

#### Disease (Metachronous Metastasis)-Free Survival and Clinicopathological Findings in the Stratified Subgroups

**Figure 3:**
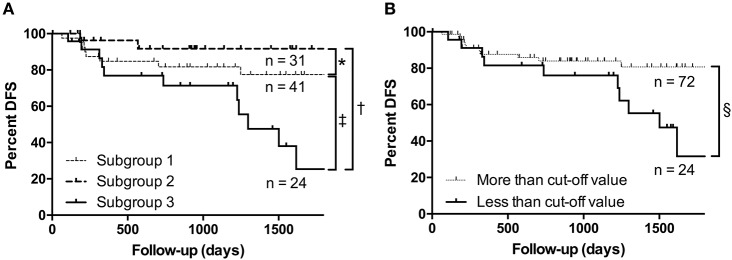
Kaplan–Meier analyses. **(A)** The rate of metachronous metastasis according to the miRNA expression subgroup. **(B)** Association between the presence of metachronous metastasis and the expression level of miRNA-135a-5p. *P < 0.471; ^†^*P* < 0.003; ^‡^*P* < 0.018; §*P* < 0.016.

The rate of metastasis-free cases was 77.1% (74 of 96 ccRCCs). Kaplan–Meier analysis was performed to determine and compare disease (metachronous metastasis)-free survival according to each miRNA expression subgroup. The results showed that the presence of metachronous metastasis was associated with the miRNA expression pattern of subgroup 3 (Figure 3A). Cox proportional hazards analysis was performed to determine and compare the disease-free survival rates. We asked whether the clinicopathological findings and stratified subgroups were independent predictors of patient disease-free survival. Toward that end, we used a univariate analysis for preliminary screening of the variables (Table 4A). This analysis was in turn followed by application of a Cox proportional hazards model. The univariate analysis (Table 4A) identified the following five factors as having an association with an increased rate of metachronous metastasis in patients with ccRCC: Fuhrman grade, necrosis, venous invasion, pT stage, and the miRNA expression subgroup obtained by the cluster analysis. These five factors may be significant predictors of the development of metachronous metastasis.

**Table 4A:** Univariate analysis of clinicopathological findings and miRNA expression patterns as predictors of metachronous metastasis in clear cell renal cell carcinoma.

**Risk factor**	**Hazard ratio**	**95% CI**	***P-*value**
**Fuhrman grade**			
3 vs. 2	2.918	1.254–6.939	0.013^*^
**Necrosis**			
Presence vs. absence	3.002	1.283–7.036	0.012^*^
**Venous invasion**			
Positive vs. negative	2.872	1.083–6.919	0.035^*^
pT stage^a^			0.029
pT2 vs. pT1	3.198	1.084–8.647	0.036^*^
pT3 vs. pT1	3.134	1.053–8.565	0.040^*^
pT3 vs. pT2	0.979	0.301–3.186	0.972
miRNA expression subgroup^b^			0.013
2 vs. 1	0.673	0.146–2.357	0.552
3 vs. 1	3.198	1.261–8.598	0.014^*^
3 vs. 2	4.746	1.476–21.041	0.007^*^

Overall significance of

a P = 0.029 and

b *P = 0.013. CI, confidence interval*.

* *P < 0.05*.

Two factors were identified in the multivariate Cox proportional hazards analysis (Table 4B). Tumor subgroup classifications (subgroup 3 vs. 2; subgroup 3 vs. 1) remained significant predictors of disease-free survival, even after controlling for the other variables. pT stage was not a factor contributing to metachronous metastasis after adjusting for the effects of the other factors. Table 4 summarizes those results.

**Table 4B:** Multivariate analysis of clinicopathological findings and miRNA expression patterns as predictors of metachronous metastasis in clear cell renal cell carcinoma.

**Risk factor**	**Hazard ratio**	**95% CI**	***P*-value**
**Fuhrman grade**			
3 vs. 2	2.217	0.812–6.100	0.118
**Necrosis**			
Presence vs. Absence	1.095	0.297–3.709	0.886
**Venous invasion**			
Positive vs. Negative	3.754	0.430–26.694	0.225
pT stage^a^			0.223
pT2 vs. pT1	2.401	0.647–7.945	0.179
pT3 vs. pT1	0.626	0.068–5.608	0.680
pT3 vs. pT2	0.260	0.040–2.179	0.207
Subgroup^b^			0.035
2 vs. 1	0.645	0.128–2.544	0.543
3 vs. 1	2.884	1.045–8.505	0.040^*^
3 vs. 2	4.471	1.250–21.544	0.020^*^

Overall significance of

a P = 0.223 and

b *P = 0.035. CI, confidence interval*.

* *P < 0.05*.

### Dysregulation of Individual miRNAs

We found that miRNA-135a-5p was an independent factor for differentiating each subgroup, as shown in Figure 2. Kaplan–Meier analysis was performed to compare disease-free survival (in terms of metachronous metastasis) according to miRNA-135a-5p expression and showed that lower expression of miRNA-135a-5p was correlated with the presence of metachronous metastasis (Figure 3B). In univariate analysis, multiple factors, including downregulation of miRNA-135a-5p, Fuhrman grade, tumor necrosis, vascular invasion and pT stage were correlated with metachronous metastasis (Table 5A). Of those, only downregulation of miRNA-135a-5p remained in multivariate analysis (Table 5B). Consequently, a cutoff value for miRNA-135a-5p higher than-1.735 determined by real time PCR was regarded as negative. The data are depicted in Tables 5A,B.

**Table 5A:** Univariate analysis of clinicopathological findings and expression of miR-135a-5p as predictors of metachronous metastasis in clear cell renal cell carcinoma.

**Risk factor**	**Hazard ratio**	**95% C.I**.	***P-*value**
**Fuhrman grade**			
3 vs. 2	2.918	1.254–6.939	0.013^*^
**Necrosis**			
Presence vs. Absence	3.002	1.283–7.036	0.012^*^
**Venous invasion**			
Positive vs. Negative	2.872	1.083–6.919	0.035^*^
pT Stage^*†*^			0.029
pT2 vs. pT1	3.198	1.084–8.647	0.036^*^
pT3 vs. pT1	3.134	1.053–8.565	0.040^*^
pT3 vs. pT2	0.979	0.301–3.186	0.972
**Expression of** ***miRNA-135a-5p***			
Low (≤ cutoff value) vs. high (> cutoff value)	3.144	1.283–7.710	0.013^*^

† *Overall significance of P = 0.029. CI, confidence interval; miRNA, microRNA*.

* *P < 0.05*.

**Table 5B:** Multivariate analysis of clinicopathological findings and miR-135a-5p expression as predictors of metachronous metastasis in clear cell renal cell carcinoma.

**Risk factor**	**Hazard ratio**	**95% C.I**.	***P-*value**
**Fuhrman grade**			
3 vs. 2	1.844	0.700–4.816	0.211
**Necrosis**			
Presence vs. Absence	1.336	0.409–4.070	0.620
**Venous invasion**			
Positive vs. Negative	3.394	0.365–24.689	0.272
pT Stage^*†*^			0.263
pT2 vs. pT1	2.110	0.596–6.518	0.231
pT3 vs. pT1	0.551	0.058–5.316	0.609
pT3 vs. pT2	0.261	0.039–2.289	0.217
**Expression of** ***miRNA-135a-5p***			
Low (≤ cutoff) vs. high (> cutoff)	2.857	1.093–7.535	0.032^*^

† *Overall significance of P = 0.263. CI, confidence interval; miRNA, microRNA*.

* *P < 0.05*.

## Measurement of the Sensitivity and Specificity of Clear Cell Renal Cell Carcinoma

We determined the cutoff value of miR-135a-5p to assess whether this marker predicted metachronous metastasis in ccRCCs. The selection of cutoff scores for individual miRNAs was based on ROC curve analysis (Supplementary Figure 1). At each expression level, the sensitivity and specificity for the outcome (metachronous metastasis) under study was plotted, thus generating an ROC curve. If an ROC curve was generated from the pairs of weighted mean sensitivities and mean specificities, then discrimination of the program for the presence or absence of the prediction of metachronous metastasis was expressed by the area under the curve (Supplementary Figure 1). The statistical package used was JMP (SAS Institute, Carey, NC, USA). As a result, miR135a-5p was the best predictor of the miRNAs that we examined (AUC 0.675; cutoff value, −1.735) in ccRCC. Consequently, < −1.735 of miRNA-135a-5p determined by real time PCR was regarded as positive.

In the present study, the sensitivity and specificity of miRNA-135a-5p were 45.5 and 81.1%, respectively. In addition, the positive predictive value and the negative predictive value were 41.7 and 83.3%, respectively. The data are depicted in Table 6.

**Table 6:** Prediction of metachronous metastasis based on the cutoff expression levels of specific microRNAs in patients with clear cell renal cell carcinoma: sensitivity, specificity, positive predictive value and negative predictive value.

**microRNA**	**Sensitivity (%)**	**Specificity (%)**	**PPV (%)**	**NPV (%)**
miRNA-155-5p	63.6	55.4	29.8	83.7
miRNA-122-5p	100.0	17.6	26.5	100.0
miRNA-21-5p	63.6	51.4	28.0	82.6
miRNA-185-3p	9.1	89.2	20.0	76.7
miRNA-106a-5p	45.5	39.2	18.2	70.7
miRNA-106b-3p	27.3	64.9	18.8	75.0
miRNA-34b-3p	77.3	31.1	25.0	82.1
miRNA-210-3p	77.3	32.4	25.4	82.8
miRNA-141-3p	72.7	20.3	21.3	71.4
miRNA-200c-3p	9.1	82.4	13.3	75.3
miRNA-135a-5p	45.5	81.1	41.7	83.3
miRNA-30a-5p	59.1	52.7	27.1	81.3
miRNA-218-5p	50.0	74.3	36.7	83.3
miRNA-429	36.4	75.7	30.8	80.0
miRNA-200a-3p	50.0	48.6	22.4	76.6
miRNA-200b-3p	90.9	18.9	25.0	87.5

*PPV, positive predictive value; NPV, negative predictive value. miR, microRNA*.

## Discussion

The identification of novel biomarkers that could predict patient outcome or therapeutic efficacy of targeted drugs is of general interest in renal carcinogenesis (7, 9). Despite the development of several biomarkers for ccRCC (the most common histological subtype of RCC), it would be helpful to have better molecular markers to reliably guide the management of ccRCC patients (32). Recently, dysregulation of miRNAs has gained attention in RCC as well as other cancers (12). In the present study, we stratified the expression patterns of 16 miRNAs that are frequently detected in ccRCCs to identify the expression pattern of miRNAs significantly associated with ccRCC. In addition, we examined the associations between the miRNA expression patterns and clinicopathological findings including development of metachronous metastasis. Finally, we attempted to identify novel miRNAs, among the 16 evaluated in this study, that are predictive of patient outcomes.

Previous studies have reported that some specific miRNAs were aberrantly expressed in RCC and were closely associated with the development of ccRCC (13–15). However, due to differences in the clinical cohorts used and the molecular heterogeneity in different studies in addition to methodological difference, reproducibility is one of the major problems (33). Our current method characterizes the expression pattern of miRNAs that are frequently found in ccRCC tissue. It should provide novel information elucidating renal carcinogenesis. Moreover, the expression profile of miRNAs might classify the tumors' molecular subtypes (33). In the present study, 3 distinct subgroups could be clearly stratified in ccRCCs. Subgroup 1 was characterized by upregulation of miRNA-155-5p, miRNA-122-5p, miRNA-21-5p, miRNA-185-3p, miRNA-106a-5p, miRNA-106b-3p, miRNA-34b-3p and miRNA-210-3p. In contrast, subgroup 2 was closely associated with downregulation of miRNA-141-3p, miRNA-200c-3p, miRNA-30a-5p, miRNA-218-5p, miRNA-429, miRNA-200a-3p and miRNA-200b-3p between subgroups 1 and 3 or 2 and 3. These findings suggest that the majority of miRNAs *in vivo* are co-regulated, and consequently form specific subgroups that could define molecular subtypes in human RCC. This finding suggests that subgroup 3, which was stratified by the expression pattern of miRNAs we used, is a novel expression profile capable of predicting metachronous metastasis of ccRCC.

In the present study, we found that the specific miRNA that makes subgroup 3 unique is miR-135a-5p. Consequently, we suggest that mir-135a-5p can be a potential biomarker in ccRCC. Therefore, we attempted to establish a quantitative cutoff value for miRNA-135a-5p. In the present study, we could show good specificity (81.1%) of miRNA-135a-5p in ccRCC we examined. This finding suggested that the cutoff value of >-1.735 for miRNA-135a-5p may predict metachronous metastasis of ccRCC. In addition, the current finding might be useful to avoid additional unnecessary treatment (adjuvant therapy) for ccRCC in clinical practice.

Recent studies have shown that miRNA-135a-5p functions as a tumor suppressor in malignant glioma and RCC (28). This hypothesis is supported by the finding that miRNA 135-a-5p is frequently downregulated in these tumors and that expression of miRNA-135a-5p is inversely correlated with pathological grading (28). In the human genome, miRNA-135a-5p maps to 2 different chromosomes: miRNA-135a-1 at 3p31.1 and miRNA-135a-2 at 12q23.1. Nonetheless, the mature RNA sequences from these two loci are identical (28). It is well known that the mechanisms that regulate miRNA-135a-5p expression differ according to cancer cell type (28). However, it is difficult to explain the contradictory effects of aberrant expression of miRNA-135a-5p in different cancer cell types. Several mechanisms by which miRNA-135a-5p could be involved in the more aggressive behavior of RCC were suggested (28). For example, it was postulated that miRNA-135a-5p might be closely associated with mitochondria-dependent apoptosis targeting *STAT6, SMAD5*, and *BMPR2* (28). Inhibition of mitochondria-dependent apoptosis might induce tumor aggressiveness. In addition, it was suggested that miR-135a-5p may target *c-MYC* (28). It is well known that *c-Myc* plays a major role in tumor invasion and metastasis (28). It was shown that *c-Myc* overexpression drives RCC in a mouse model through glutamine metabolism, because *c-Myc* and glutaminase are components of glutamine metabolism that are overexpressed in human RCC (34). However, an opposite finding was suggested by data that indicated that miRNA-135-a-5p functioned as an oncogene in colorectal cancer, targeting the tumor suppressor *APC* (35). In the present study, dysregulation of miRNA-135a-5p was well correlated with metachronous metastasis, suggesting miRNA-135a-5p may be a potential prognostic biomarker in ccRCC.

A recent study has shown that miR expression is not correlated with host genes or primary miRNA transcripts, indicating that post-transcriptional regulation of miR expression occurs in a subset of human cancers (36). It has been reported that the expression level of miR is regulated by *DROSHA* in human cancer (37). In addition, a recent study showed that downregulating *DICER1* expression that is also associated with regulation of miRNA transcripts promotes tumorigenesis *in vitro* and in a mouse lung cancer model (38). According to this theory, expression of *DROSHA* and *DICER1* might play important roles in the function of specific miRs in human cancers *in vivo*. Unfortunately we could not examine the expression of the two components in the present study. Nonetheless, it is possible that in renal carcinogenesis, an association could exist between the expression of such components and dysregulation of specific miRs.

There are some limitations to this study. First, the number of patients enrolled in the study was generally small. Comparing comprehensive “big data,” such as those in TCGA (13, 14), with data from an individual study is important to develop diagnostic and prognostic markers for cancers such as ccRCC. Second, in retrospective cohort studies, a second cohort for validation purposes, in addition to the first cohort, may be necessary to identify the outcomes of patients with ccRCC. The present study, however, was limited to a single cohort. In the near future, we plan to validate our results presented here. Third, in the present study, we could not compare miRNA expression patterns between primary RCC and metastatic cancer tissues. Such information could be valuable; however, it is difficult to obtain frozen tissue derived from metastatic lesions. Finally, the miRNAs evaluated might be selected arbitrarily, which may have introduced selection bias. Although high-throughput analysis using microarrays has been performed in previous studies, here we evaluated 16 miRNAs that are closely associated with renal carcinogenesis. However, the selection of these specific miRNAs would be greatly supported by adding specific references implicating each miRNA in ccRCC (17, 18, 20–32). Despite the selection of these miRNAs, we showed that the rate of metachronous metastasis was associated with a specific expression pattern of these miRNAs, and that lower expression of miRNA-135a-5p was correlated with metachronous metastasis. We believe that our findings pertaining to these select miRNAs provide new insight into the mechanism of renal carcinogenesis.

In conclusion, we examined the expression pattern of multiple miRNAs in ccRCC using cluster analysis and found that they could be stratified into 3 distinct subgroups. In addition, the present findings showed that subgroup 3, which is characterized by downregulation of miRNA-135a-5p, is useful for the prediction of metachronous metastasis of ccRCC in multivariate analysis. Finally, a cutoff value of miRNA-135a-5p was set to determine whether such a value could be useful to predict metachronous metastasis in ccRCC. We suggest that a cutoff value of miRNA-135a-5p helps to predict metachronous metastasis of ccRCC. Further study is needed to identify the role of miRNAs in renal carcinogenesis.

## Ethics Statement

Written informed consent regarding tissue specimens for research purposes was obtained in each case. This study was approved by the Iwate Medical University (Iwate Medical University Ethical Committee; HGH29-25) and was conducted in accordance with the Helsinki Declaration. All procedures were performed in accordance with the ethical standards of Iwate Medical University and with the Declaration of Helsinki. An alternative of informed consent (approved by the Institutional Review Board of Iwate Medical University) was obtained from all patients included in the study.

## Author Contributions

ES is the first author, constructed the figures and tables and performed statistical analyses. TS is the corresponding author, contributed to the preparation of the manuscript and all aspects of data collection and analysis. KI and MO performed histological diagnosis. TT, YK, RT, and WO assisted with clinical data.

### Conflict of Interest Statement

The authors declare that the research was conducted in the absence of any commercial or financial relationships that could be construed as a potential conflict of interest.

## Supplementary Material

Supplementary Figure 1 ROC curves of examined microRNAs in clear cell renal cell carcinoma. **(A)** miRNA-155-5p; **(B)** miRNA-122-5p; **(C)** miRNA-21-5p, **(D)** miRNA-185-3p; **(E)** miRNA-106a-5p; **(F)** miRNA-106b-3p; **(G)** miRNA-34b-3p; **(H)** miRNA-210-3p; **(I)** miRNA-141-3p; **(J)** miRNA-200c-3p; **(K)** miRNA-135a-5p; **(L)** miRNA-30a-5p; **(M)** miRNA-218-5p; **(N)** miRNA-429; **(O)** miRNA-200a-3p; **(P)** miRNA-200b-3p.
