# Supplementary figures and images for: Analysis of Expression Patterns of MicroRNAs That Are Closely Associated With Renal Carcinogenesis
Source: Front Oncol. 2019 May 31;9:431. doi: 10.3389/fonc.2019.00431 (PMC6555129; doi:10.3389/fonc.2019.00431)

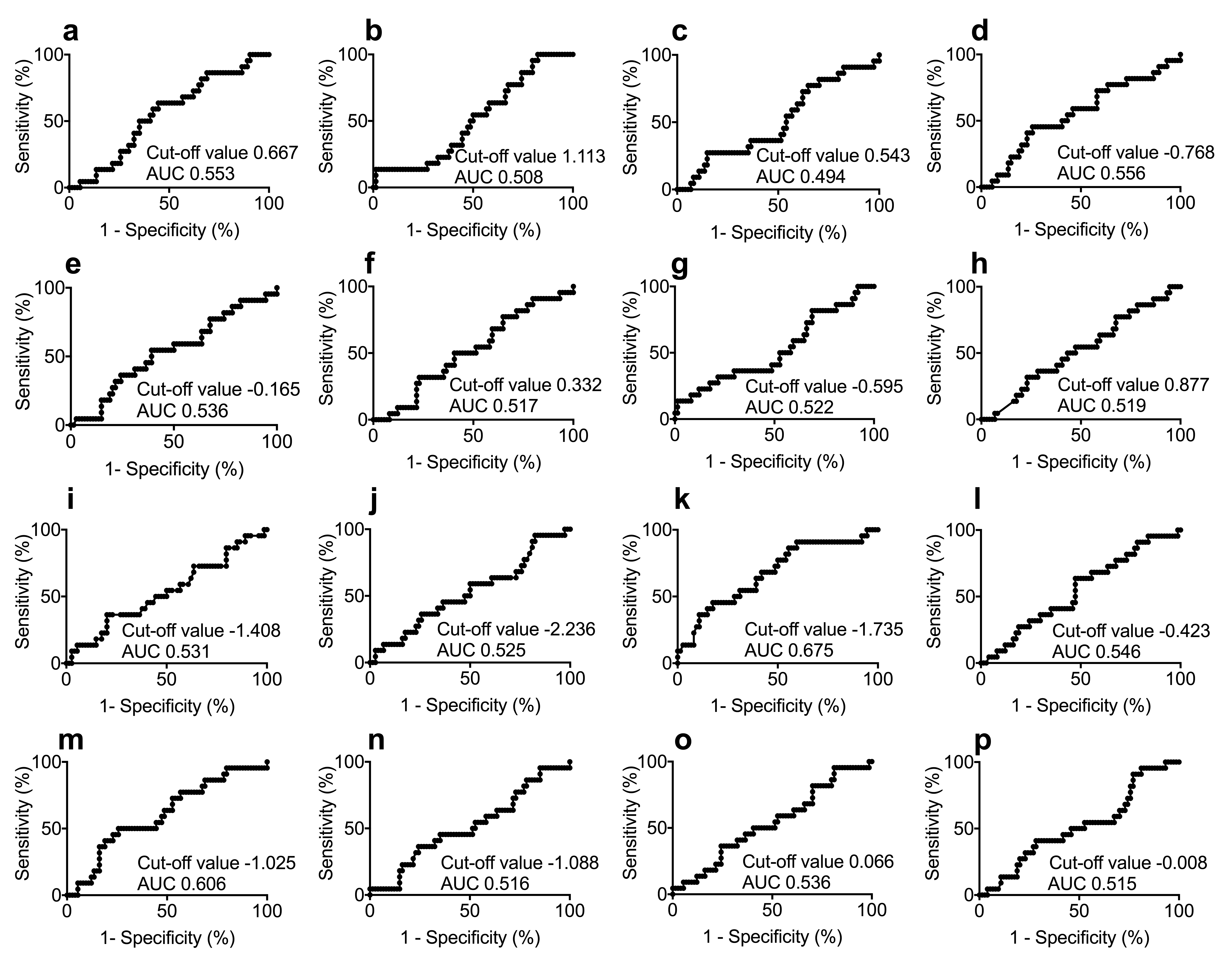

Supplement: Supplementary Figure 1 — ROC curves of examined microRNAs in clear cell renal cell carcinoma. (A) miRNA-155-5p; (B) miRNA-122-5p; (C) miRNA-21-5p, (D) miRNA-185-3p; (E) miRNA-106a-5p; (F) miRNA-106b-3p; (G) miRNA-34b-3p; (H) miRNA-210-3p; (I) miRNA-141-3p; (J) miRNA-200c-3p; (K) miRNA-135a-5p; (L) miRNA-30a-5p; (M) miRNA-218-5p; (N) miRNA-429; (O) miRNA-200a-3p; (P) miRNA-200b-3p. [file Image_1.tiff]
